# Obligations of low income countries in ensuring equity in global health financing

**DOI:** 10.1186/s12910-015-0055-3

**Published:** 2015-09-08

**Authors:** John Barugahare, Reidar K. Lie

**Affiliations:** Department of Philosophy, University of Bergen, 5020 Bergen, Norway

## Abstract

**Background:**

Despite common recognition of joint responsibility for global health by all countries particularly to ensure justice in global health, current discussions of countries’ obligations for global health largely ignore obligations of developing countries. This is especially the case with regards to obligations relating to health financing. Bearing in mind that it is not possible to achieve justice in global health without achieving equity in health financing at both domestic and global levels, our aim is to show how fulfilling the obligation we propose will make it easy to achieve equity in health financing at both domestic and international levels.

**Discussion:**

Achieving equity in global health financing is a crucial step towards achieving justice in global health. Our general view is that current discussions on global health equity largely ignore obligations of Low Income Country (LIC) governments and we recommend that these obligations should be mainstreamed in current discussions. While we recognise that various obligations need to be fulfilled in order to ultimately achieve justice in global health, for lack of space we prioritise obligations for health financing. Basing on the evidence that in most LICs health is not given priority in annual budget allocations, we propose that LIC governments should bear an obligation to allocate a certain minimum percent of their annual domestic budget resources to health, while they await external resources to supplement domestic ones. We recommend and demonstrate a mechanism for coordinating this obligation so that if the resulting obligations are fulfilled by both LIC and HIC governments it will be easy to achieve equity in global health financing.

**Summary:**

Although achieving justice in global health will depend on fulfilment of different categories of obligations, ensuring inter- and intra-country equity in health financing is pivotal. This can be achieved by requiring all LIC governments to allocate a certain optimal per cent of their domestic budget resources to health while they await external resources to top up in order to cover the whole cost of the minimum health opportunities for LIC citizens.

## Background

The Alma Ata Declaration (1978) categorically stated that “the existing gross inequality in the health status of the people particularly between developed and developing countries as well as within countries is politically, socially and economically unacceptable and is, therefore, of common concern to *all* countries” (emphasis added) [1]. Following this declaration there has been wide recognition of *shared responsibilities for global health* implying that all countries – High Income Countries (HICs), Middle Income Countries (MICs) as well as LICs – have a joint responsibility for improving global health with equity as one of the overarching goals. However, currently the actual proposals regarding obligations of countries to contribute to efforts towards global health equity largely ignore specific obligations of LIC governments, particularly obligations relating to health financing.^1^ The negligible attention given to LIC governments’ obligations is evident in a number of global initiatives aiming at improving health opportunities of citizens in poor countries. Some of these initiatives include the proposed Framework Convention on global health [2]; the Global plan to combat neglected tropical diseases [3]; and the Global strategy and plan of action on public health, innovation and intellectual property [4]. Further, the findings and recommendations of the Commission on intellectual property rights, innovation and public health [5]; the Report of the consultative expert working group on *Research and Development: Financing and Coordination* [6] among others are all concerned with international health resource transfer from HICs to LICs. So far it seems to be taken for granted that LIC governments are doing everything possible within their limited resource contexts to fulfill the health needs of their citizens. But whereas it is necessary to mobilize external resources to fill the health-resource-gaps in LICs, a lot of evidence has suggested that there is need to think about possible obligations of LICs themselves in health financing [7–13]. All these authors point at various weaknesses in health financing within LICs which cause health inequities within those countries. It is also important to note that these domestic inequities are usually reproduced in the general global health picture whenever international health comparisons are made. With very good reasons all the above authors strongly imply that most LIC governments behave as if they are *unwilling* rather than *unable* to equitably fulfil the health needs (or rights) of their citizens through, among other things, equitable health financing. Drawing from these views our argument is that these weaknesses in health financing within LICs can be significantly reduced if LIC governments fulfill certain obligations relating to health financing. We argue and recommend that LICs should bear an obligation to allocate a certain minimum percentage of their domestic resources to health, while external resources should be used to top up whatever shortfall remains in order to achieve a certain minimum level of health opportunities for all individuals. For the purpose of implementing the obligation we are proposing we will recommend a specific mechanism for reducing intra-country inequities and show that this will at the same time reduce inequities in inter-country health financing.

After some conceptual clarifications we begin by providing evidence of low priority given to health sectors in LICs’ budget allocations, and we later use this evidence as a basis for defending the view that in the pursuit of global health equity LIC governments should fulfill the obligation we are proposing. We then begin our main discussion by stating and explaining the obligation of LICs which we are advocating. We go ahead to recommend a mechanism which helps to reveal, understand and mitigate the existing inequities in health financing between and within LICs as well as between all countries rich and poor. We demonstrate the effectiveness of this mechanism in determining LIC and HIC governments’ fair and optimal financial obligations for health financing and go ahead to show how fulfilling the resulting obligations by each country will lead to equity in global health financing as one of the steps towards global health justice.

### Underlying concepts

In this paper we shall understand *health inequity* in general as undeserved inequalities in health status or access to health services which can be reduced by human efforts. By *global health inequity* we mean health inequity among individuals irrespective of national borders, or health inequities between and within countries. *Equity in global health financing* will be understood *here* as an inter- and intra-country proportionate distribution of the financing burden for *a certain minimum* level of health opportunities for all individuals globally by taking into account differences in resource capacities of different countries. By *Global Minimum Health Expenditure (GMHE) per capita* we mean the average cost of financing an ‘Essential Health Package’ per person per year in each country, or as we call it, ‘a certain minimum level of health opportunities per capita’. Our concept of the “minimum” follows from the WHO concept of an ‘Essential Health Package’ which is supposed to be *a guaranteed minimum* of “a limited list of public and clinical interventions which will be provided at primary and/or secondary level care” for citizens of LICs (emphasis in original) [14]. Other ideas which point to the concept of the “minimum” include “Universal Coverage” for all people which “does not necessarily mean coverage for everything …” [15]; the ‘progressive realization of the right to health’; ‘Essential Medicines/Drugs’ among others. All these concepts imply that there is a certain minimum of health opportunities that should be seen as *a right* of every individual globally as recommended by the Alma Ata Declaration’s concept of Primary Heath Care [16]. The reasoning in our discussion reflects a view that if individuals are not assisted to realise this moral right yet it is within the means of obligation bearers (both HIC and LIC governments), then such obligation bearers are acting unjustly. Since we propose that the cost of covering this minimum be shared equitably between HICs and LICs (taking into account the resource capacity of each of them) then if such a minimum is not covered, then it will be possible to identify the source of injustice by looking at which actor(s) refused to fulfill their quota of obligation. But most importantly our concern in this paper is that the minimum health opportunities should be fully and equitably financed by *public resources* from both LICs and HICs without any private health expenditure (PHE). Therefore, in our discussion *Global Health Justice* is, or consists in, guaranteeing a certain minimum level of health opportunities to all individuals globally and ensuring an inter- and intra-country equitable sharing of the burden to that effect.

### Evidence of low priority given to health sectors in LIC budgets

There are suggestions that LIC governments allocate a lesser percentage of their annual domestically generated budget resources to health than they should, and could afford. For instance, considering the World Health Organisation (WHO) Africa Region, Africa’s annual average budget allocation to health as a percentage of total government budgets is 8.7 % compared with Europe’s 14.8 % and the Americas’ 16.8 % [17]. By the year 2001 the African Heads of States and Governments of African Union member states had realised that they were allocating negligible percentages of their budgets to health amidst increasing amounts of resources needed to respond to the HIV/AIDS epidemic, Tuberculosis and other communicable diseases. Having realised these financing gaps in the health sectors across Africa due to low priority given to health budgets, they all committed themselves to allocating at least 15 % of their annual budgets to health in what they called the *Abuja Declaration on HIV/AIDS, Tuberculosis and other Related Infectious Diseases* [18]. However, since 2001 few African countries have reached this target; and currently the trend is regressing. For example, the WHO revealed that “19 of the countries in the region who signed the declaration allocate less now than they did in 2001” [19]. Only four countries (Rwanda, Botswana, Zambia and Togo) fulfilled their commitment to the Abuja declaration’s 15 % [20].

In reaction to this relapsing trend, there is a view that Africa needs to improve on the laudable Abuja commitment and progress from “just 15 % to ‘15 % plus” by doubling per capita investment in health, and also more in crucial social determinants and pillars of health [21]. Further, the WHO has recommended that increased priority should be given to health from the general budget and/or debt relief funds. From the analysis undertaken for the World Health Report of 2011, “it is clear that some countries need to increase their own investments in health either through reallocation within their own general budgets or by making larger claims on their funds from debt relief which are to be preferentially allocated to social spending” [22]. For example, in Uganda it has been observed that the health sector is unnecessarily neglected and as a consequence the Uganda health sector has been found to be in a very sorry state, unnecessarily [23–25]. But despite repeated pleas with the government to increase resources to the health sector, “the government has continuously shown unwillingness to make the health sector its priority in budget allocations” [26].

This alleged avoidable lack of sufficient funding for health within developing countries by the LICs themselves is the background for our argument that in addition to discussing obligations of HIC governments to, among other things, transfer health resources to LMICs, complementary obligations of the latter should also be emphasised. This is because from the point of view of fairness – as it has been argued in the case of human rights obligations – “a sound case for transnational obligations cannot be made, intellectually or politically” [and in this case morally], without eventually defining the scope and limits of national obligations” [27]. But most importantly as our view is that it will remain extremely difficult to achieve justice in global health unless obligations of LICs are identified and implemented.

## Discussion

### LICs’ minimum financial obligation for health

The obligation of LIC governments we are arguing for is that all LICs should allocate a certain uniform minimum percentage of their domestic budget resources to their health sectors as their contribution to cover part of the cost of the minimum health opportunities for each of their citizens. In this paper we do not intend to recommend the *actual* percentage of domestic budgets which all LICs should allocate to their health sectors. This is because such a recommendation will have to be evidence-based taking into account the resource capacities of different countries and other relevant factors, all of which are beyond the means and resources of this paper. However, we will use a hypothetical percentage and then proceed to recommend and demonstrate a mechanism for dividing proportionately between HIC and LIC governments, the total cost (financing burden) of meeting these minimum health opportunities and this mechanism takes as its point of departure the optimal size of LIC governments’ obligation.

In order to get to the root of the obligation we are proposing for LIC governments and how it will ensure equity in global health financing, we will take as our point of departure the estimate provided by the WHO of the cost of resources needed to provide basic life-saving services (health opportunities) per person per year in developing countries which is US$ 44 per person per year [28]. The obligations of LIC governments and external governments should be specified in terms of percentages of this cost, beginning with an optimal size of LIC governments’ obligations. This implies that all countries which need external health resources in order to achieve the targeted minimum of health opportunities (expressed as GMHE per capita) should allocate a certain specific optimal percentage of their domestically generated annual budget resources to their health sectors in order to fulfill their quota of health financing obligation. Basing on the deficits that will remain after fulfilling this (LIC) obligation it will make it easy to determine the exact size of the obligations of external governments.

For its effectiveness, this obligation presumes and recommends coordination in global health financing especially in international health resource transfers. Coordination is crucial because it has been observed that uncoordinated health financing between countries has no capacity to achieve global health equity and at the same time it has a high potential of deepening the existing inequities [29]. However, coordination in financing global health partly requires a mechanism for determining international obligations owed to each country, particularly those in need of external resources to cover the minimum health opportunities per capita. But again as a point of emphasis, in order to determine the needed resources from HICs to LICs it is important first of all to determine an optimal level of health financing that can be reasonably expected from LICs by virtue of their resource capacities. It is this level that should constitute their (LICs) obligation, while the deficit that remains constitutes the size of external obligations. Fulfilling these obligations will lead, *at least in principle*, to inter- and intra-country equity in financing the global minimum of health opportunities that should be guaranteed to all individuals globally as a matter of right. For the purpose of implementing this obligation we recommend and illustrate a mechanism proposed by Ooms and Hammonds [30] for determining the size of HIC obligations in fulfilling the right to health for citizens in LMICs. We will later illustrate how fulfilling the resulting obligations leads to equity in financing global health.

#### A mechanism for achieving equitable burden-sharing in global health financing

The point of departure for Ooms and Hammonds is a challenge set by Norman Daniels in his book *Just Health* [31]. After defending the ethical appropriateness of shared responsibilities for health at a national level, Daniels expresses pessimism at the possibility of extending the ethical principles he used to a global level [30] and sets this as challenge for those interested in global health justice. In *Taking up Daniels’ Challenge*, Ooms and Hammonds propose a mechanism for determining the amount of financial resources HICs should transfer to LMICs in order to realise the right to health.^2^ It should be noted that whereas Ooms and Hammonds’ emphasis is on HIC obligations to ensure the fulfilment of the right to health in LMICs, our emphasis is on the obligations of LICs themselves in ensuring global health justice, particularly equity in global health financing. Their mechanism *implies* that determining a reasonable size of financial obligations of LIC governments entails that what remains is a fair size of HIC obligation. Hence, whereas Ooms and Hammonds use this mechanism in their argument for, and illustration of how to determine *HIC obligations to ensure the right to health*, we will adopt this mechanism in our argument for, and illustration of, how determining and implementing LIC obligations will ensure equity in global health financing.

Ooms and Hammonds show that given that the amount of resources needed for each individual to ensure a just level of health [opportunities] is known, if all LICs allocated a certain minimum percentage of their GDP to health, it would be easy to determine the deficit for each individual in that country in order to reach the targeted minimum of such health opportunities in form of GMHE per capita’. In turn the total of these individual deficits would constitute the size of international health assistance owed to each poor country. Their hypothetical illustration is summarised as follows:

- If [poor] Country A has a GDP per capita of about US$333 and commits 3 % of this amount, or US$10 per person per year on the distribution of health related goods, then the global obligation towards country A is limited to ensuring that it can achieve health-related goods distribution worth US$40 per person per year, assuming that this financing level [US$40] is what it takes to realize the core content of the right to health. Hence the international obligation to such a country would be an equivalent of US$30 per person per year. And if Country B has a GDP per capita of US$1,000, and commits 3 % of that amount (or US$30 per person per year) on health-related goods, the global responsibility towards B would be the equivalent of US$ 10 per person per year. Then, if Country C has a GDP per capita of US$2,000, and allocates 3 % of that amount (or US$60 per person per year) on the distribution of Health-related goods, then there would be no global responsibility towards C [30].

This mechanism implies that once the obligations of LICs we are proposing are fulfilled, the size of each LIC’s claim on international health resources will be directly proportional to its level of poverty. That is to say, poorer countries will be morally entitled to more health-aid per capita compared to countries which are better-off (such as MICs). This will ensure proportionate sharing of the burden of financing the targeted level of health opportunities for all individuals globally. It will mean that this minimum level of health opportunities is 100 % publicly financed from contributions of domestic governments and external governments. At this point there will be no financial barriers to such minimum health opportunities at the point of service delivery as well as financial inequities between individuals which normally arise from private health expenditures and more especially Out of Pocket Payment (OPP).

Generally, what Ooms and Hammonds have done is to show that it is possible to provide a reasonable way of specifying and coordinating international obligations to provide health aid. Below we show how, basing on our proposed obligation, this framework can be used to identify lack of equity in health financing between LICs themselves and between HICs and LICs in their effort to meet the health needs of LIC citizens.

#### Some preliminary points

First, whereas Ooms and Hammonds use the percentage of GDP in illustrating the mechanism for determining the size of HIC obligations, in our illustrations we will use the percentage of *domestically generated annual budget resources*. This is because the percentage of GDP devoted to health is much broader. GDP does not usually indicate the actual public resources available for health spending in a given financial year, and it also includes private contributions (PHE) which we seek to avoid in financing the targeted minimum of health opportunities. Therefore, since our concern is with the actual amount of public resources that can be made available from a certain percentage of the budget, instead of using *‘GDP per capita’* we will use *‘domestic annual budget per capita’.* We will exemplify our illustrations by considering health financing in two countries from the WHO Africa region – Uganda and Kenya. In subsequent examples we will use a few more countries in the same region that receive external health resources. Secondly, whereas the WHO estimates the average cost of basic life-saving services per person per year at US$44, the actual cost in different countries will obviously vary depending on the specific needs of each country, local costs of services among others. This means that in some countries the actual cost will be less than, and in some others above, US$44. In our illustrations we use a higher *hypothetical* figure of US$60 in order to account for these variabilities and the potential need for the minimum health opportunities to go beyond simply life-saving services. But whatever the actual cost per country may turn out to be, it should depend on evidence from country-specific studies. And in order to ease our illustrations we shall use a uniform cost of the minimum services (US$60) in all our examples since this does not affect the validity of the obligation we are proposing and the mechanism we are recommending.

#### Current inequities in health financing between countries

Table 1 demonstrates the current inequity in health financing between Uganda and Kenya as a result of the different percentages of their domestically generated annual budget resources allocated to health.

**Table 1:** Inequitable health financing due to unequal budget allocations to health between Uganda and Kenya

Country	Total domestic budget per capita (US$)	Health expenditure as % of total budget per capita	Absolute health expenditure per capita (US$)	Global minimum Health Exp. (GMHE) (US$)	Deficit to reach GMHE per capita (US$)
Uganda	152	7.4	11.25	60	48.75
Kenya	226	5.4	12.20	60	47.80

**Source:** Constructed on the basis of figures from Uganda’s Health Sector Performance Report, 2012/13 and Kenya National Health Accounts 2009/10

Total Budget Per Capita has been calculated using Actual Health Expenditure (from public domestic resources) for each country as a percentage of total domestic budgets (without external funds)

Table 1 shows that Kenya’s Total Budget per capita for 2009/10 (excluding aid) was around US$226 and only 5.4 % of it was allocated to health, generating US$12.20 per person. In the case of Uganda the domestic budget was around US$152 per person, and 7.4 % of this was allocated to health leading to US$11.20 per person. This means that if we *assume* the average cost of the minimum health opportunities to be US$60 in both countries, it means that in order to reach this target Kenya will need extra US$47.80, while Uganda’s deficit will be US$48.80. But since Kenya is wealthier (higher GDP/budget per capita) than Uganda, Kenya can allocate to health a smaller percentage of its budget than Uganda to reach the same absolute per capita contribution or even higher as Table 1 shows. This implies that these two countries are carrying disproportionate burdens in financing the global minimum health opportunities. This is an example of inter-LIC inequity in global health financing.

Hence, in order to equalise the financing burden between the two countries there is need to specify an optimal percentage of domestic budget resources which all LICs should allocate to their health sectors and then proceed to calculate the shortfall for each of them needed from external health resources. This will ensure that each country’s size of claim on external resources is proportionate to its level of poverty and in relation to claims of other health-aid seekers. For example, if LIC financing obligation was to be set at Kenya’s 5.4 % (in Table 1) the whole of the current shortfalls for both countries (and even more) would become a HIC obligation. If it were set at Uganda’s 7.4 %, Uganda would receive the whole of her current shortfall from external resources, while Kenya would be entitled to less than it could claim if the minimum were set at 5.4 %. Yet, if the Abuja Declaration’s target of 15 % referred to earlier *were* to be accepted as an optimal financial obligation for LIC governments, then the resulting lower deficits to reach the GMHE per capita would entail that both countries would have to contribute additional resources to cover part of their current shortfalls, but Kenya would have to contribute proportionally more compared to Uganda given her higher GDP. In other words, by equalising these countries’ burden of health financing, their current relative claims on international resources change in favour of Uganda which currently bears a disproportionate burden in relation to Kenya. The moral essence of this point is that since justice requires proportionate distribution of burdens/costs, this change in relative claims which is in favour of Uganda is morally justified and necessary.

Earlier we mentioned that there is a widely shared view that most LIC governments allocate negligible percentages of their annual domestic budgets to health. Therefore, if this is true, then it implies that if the whole of LICs’ current shortfalls is demanded from HICs, there will be disproportionate burden sharing between LICs and HICs because LICs are imposing part of their obligation onto HICs. Hence in our next illustration we shall use a higher percentage which was recommended by the African Heads of States and governments. In the view of the African Heads of States and Governments as expressed in the Abuja Declaration of 2001, arguably, African countries have the capacity to allocate at least 15 % of their annual budgets to improving health. So, for the purpose of this illustration we will *assume* 15 % of domestically generated annual budget resources as the optimal financial obligation for LIC governments. We will use it to demonstrate how fulfilling our proposed LIC government obligation leads to inter-LIC equity in global health financing 3 and equity between HICs and LICs. However, there are a few important points to note about the Abuja target of 15 %. First, the Abuja Declaration does not specify whether 15 % is of the total government budget including external resources, or if it is a percentage of domestically generated resources only. Secondly, in the Declaration there is no independent justification of 15 % from the point of view of resource capacities of countries. Failure to take into count resource capacity might explain why most African countries have not lived up to their commitments, hence suggesting the need for an evidence-based financing target. But despite all these issues, for the *limited purpose* of our illustration we shall take this 15 % as a percent of domestically generated budget resources agreed upon by all actors.

Table 2 shows how much health aid would need to be contributed by international donors if one sets a uniform obligatory level (obligation) of health financing for LIC governments as percentage of their domestic annual budget at 15 %. For the two countries considered earlier (Uganda and Kenya) for example, their deficits will fall. In the case of Uganda it will decline from US$48.8 (in Table 1) to US$37.2 (Table 2); while for Kenya it will reduce from US$47.80 (Table 1) to US$26.1 (Table 2). This shows that the two countries would increase their respective domestic contributions by different absolute amounts as a result of increasing their budget percentages to health. Kenya would contribute an additional 21.7 US$ (US$ 33.9 – 12.2) and Uganda 11.6 US$ (US$ 22.8 – 11.2). The impact of this obligation on justice is that by doing their best in health financing, LICs will reduce the burden of health financing currently borne (or expected to be borne) by HIC citizens. In other words, on top of all LICs bearing an equal burden of financing the minimum health opportunities per capita, the resulting deficits will be a fair size of obligation for HICs since LICs will have reached a level where they are *unable,* rather than *unwilling,* to allocate more resources to health.

**Table 2:** The effect of standardising financing obligation at Abuja’s 15 % on inter-country financing equity

Country	Total budget per capita (US$)	Current domestic % of budget and resulting absolute per capita US$ (X% = US$)	Absolute US$ from 15 % of domestic resources per country	GMHE per capita (US$)	Deficit (US$) per capita to reach GMHE (US$ 60)
Uganda	152	7.4 % = 11.2	22.8	60	37.2
Kenya	226	5.4 % = 12.2	33.9	60	26.1
Rwanda	130	23.3 % = 30	19.5	60	40.5
Equatorial Guinea	6943	7.0 % = 486	1041	60	**No Deficit**
Tanzania	126	11.1 % =14	18.9	60	41.1
Namibia	2400	6.5 % = 156	360	60	**No Deficit**

**Source:** Constructed from National Health Expenditure Statistics for Uganda and Kenya (Table 1); and Estimates for the rest of the countries’ Annual domestic budget per capita has been calculated basing on the 2010 Health Expenditure Per Capita as percentage of Annual health budget (without foreign resources) Provided by Afri-Dev. Info. 2013. (Post Abuja + 12) 2013 Africa Health Financing Scorecard – Featuring Year 2000–2010 Indicative Progress Summary

Further, given that LIC governments need to increase their domestic contribution to health [15], it will be possible to save external resources that are now going to countries (LMICs) 4 that have the capacity to afford the global minimum of health opportunities per capita without international resources if such countries fulfilled their 15 % or whatever the size is of this minimum obligation will turn out to be. Such countries would be, for example, Equatorial Guinea and Namibia in Table 2, since they would have “No Deficit”. With the possibility of coordinated global health financing, especially external health financing to cover the GMHE per capita, it would be possible to redirect some of the external resources currently going to countries like Equatorial Guinea and Namibia to countries which, even after allocating 15 % or more of their domestic budgets to health, such as Rwanda, cannot raise the needed GMHE per capita estimated at US$60. The underlying moral reasoning here is that it is unjust to give assistance to countries which are, or can be, above the targeted threshold of health-opportunities without assistance before sufficient assistance is given to others which have not yet reached, and cannot reach, the same threshold without extra assistance by virtue of their more constrained resource capacities. In other words among LMICs there are some countries which are capable of fully financing the minimum health opportunities for their citizens without external assistance yet such countries are currently receiving health aid before poorer ones get what they need. Our view is that when such countries claim external resources before worse-off countries get enough to cover their minimum health opportunities, then such claims (by better-off countries) are unjust. So far this category of moral reasoning is evidently lacking in the current manner in which global health is financed, particularly international health resource transfers. This poses a serious threat to the efforts and hope of achieving global health equity.

Presently, if we go by actual health financing statistics among WHO African region Member States, in 2010 Equatorial Guinea and Namibia allocated only 7 and 6.5 % respectively of their annual domestic budgets’ resources to health and they were able to raise as high as US$486 and US$156 per person respectively; yet at the same time, between 2000 and 2010 Equatorial Guinea received a cumulative total of US$30.50 million from the Global Fund alone; while Namibia received US$187.72 million [20]. Hence, from the point of view of what constitutes just or unjust claims on external resources as described above, the amount of Global Fund resources claimed and received by Namibia and Equatorial Guinea constituted *unjust claims* on international health resources. This is especially the case because there are some economically worse-off countries which could not raise the GMHE per capita even if they allocated as high as 23 % of their annual budgets to health (Rwanda); and also the amount of health aid they received could not enable them reach the GMHE per capita of US$60. Therefore, since equitable distribution of burdens is a relevant factor in the pursuit of justice in global health, our mechanism implies that Namibia and Equatorial Guinea should not have received external health resources before Rwanda received what it needed to reach the U$60 target. In this case Rwanda and all countries which our mechanism places in a Rwanda-like situation bore a disproportionate burden compared to Namibia and Equatorial Guinea in attempting to cover the cost of the global minimum level of health opportunities. Further since the GMHE per capita for each country is expected to cover the basic life-saving services it means that citizens of Rwanda on one hand, and those of Namibia and Equatorial Guinea on the other, had unequal access to these life-saving services. This illustrates the kind of financing inequities that would be avoided if all LMIC governments were required to allocate a specific optimal percentage of their annual (domestic) budgets to health as we are proposing in respect to LICs.

Again, going by the figures provided by the *2013 African Health Financing Scorecard*, it would have been possible to save and redirect Global Funds totaling to at least US$1.02 billion from just 12 of these (African) countries. It should also be noted that this amount would have been saved at the current health financing levels (below 15 %) of these 12 countries. This implies further that instead of asking the entire deficit for the rest of LICs from the HICs, if a mechanism like this had been followed in resource fundraising and disbursement, the burden of HIC citizens could have been reduced even further and would be even fairer. At this point it is important to reiterate our disclaimer that the 15 % which we have used in this illustration does not have to be the actual size of LIC governments’ financing obligation. Rather, it has been used to show how the obligation of LIC governments to allocate a certain uniform percentage of their domestically generated budget resources to health along with mechanism we have recommended have the capacity to lead to equity in global health financing.

One objection to our argument could be that HICs should not reduce their transfers to better-off poor countries which do not fulfill their obligations in order to be able to increase their (HICs) transfers to poorer countries that are in greater need. Rather, it may be recommended in this objection that HICs should continue to support the non-compliant better-off countries and at the same time increase their transfers to poorer countries. However, this objection ignores the fact this strategy rewards non-compliance and, therefore, maintains inequities in international health financing. This is so because there is evidence that for each dollar they receive in form of health aid, some LIC governments reduce their health expenditure from their domestic resources [28, 32]. This is corroborated by the evidence provided by the *2010 African Financing Scorecard* which shows that between the year 2000 and 2010, presumably due to the Global Fund and GAVI funds effect, health financing as percentage of domestic budget resources declined in 12 African countries [20]. It is important to note, however, that the decline in domestic funding in the wake of increased external resources could also be attributed to a number of macro-economic goals especially IMF budget ceilings in LICs [33]. However, IMF recommends ceilings for general social sector spending implying that if health is prioritised within social sector budget allocations there will be no need for extra fiscal space to accommodate an increase in health budgets. But even if we work within an IMF imposed budget ceiling, this does not refute our general claim that we should agree on a level of domestic health spending, whatever that is, and use that level to work out an equitable distribution of international transfers of aid.

By and large, the illustrations above show, by the specific examples of Uganda and Kenya, that even though the burden borne by Uganda in financing health is higher than that of Kenya, Uganda still has lower health expenditure per capita from public funds than Kenya yet both countries compete for the limited external health resources without any assurance that Uganda will be given priority to Kenya in external resource (per capita) disbursements. This situation obtains between majority of LMICs. This means that the size of Kenya’s claim on international health resources is unfair in relation to that of Uganda. In relation to HICs it is an unfair claim because Kenya is doing less than it can by virtue of its resource potential for health financing. To put it differently, when LICs allocate less than what might turn out to be their optimal obligation and then claim the entire deficit from HICs they (LICs) end up unfairly imposing part of their justified burden to citizens of both HICs and other LMICs. The whole problem of lack of uniform and stringent obligations of LIC governments in financing health leads to a disproportionate sharing of the burden of financing global health leading to inequity in global health financing. However, fulfilling LIC governments’ minimum financial obligations for global health as proposed here along with the mechanism we have recommended will lead to equity in financing global health, in particular the essential (minimum) health package for all individuals globally; that is, financing equity between LMICs themselves, and between LMICs and HICs.

#### LICs’ financing obligation and intra-LIC equity

In the discussion above we have illustrated how the obligation of LIC governments to allocate a certain uniform minimum percentage of their domestic budget resources to health can be effective as a mechanism for guiding equitable health resource contributions and disbursements globally, leading to inter-country equity. Now we will illustrate how, *in principle*, this obligation leads to equity in domestic health-financing and then suggest additional obligations that would make such equity a reality.

There is ample evidence that in nearly all LICs public spending on health disproportionately benefits wealthier citizens and also that health financing is very disproportionate in favor of the rich [7, 9, 10]. This is inequity in health financing and it strongly determines how much PHE as a financing mechanism is relied on in such health systems. In some LMIC health systems PHE as a percentage of Total Health Expenditure (THE) surpasses the total contributed by both public and donor contributions to health spending.^5^ This situation obtains in the financing of all health services even beyond the minimum health packages. And given the very high income inequalities in most LICs, the obvious consequence of high dependency on PHE, especially out of pocket payments, is that poorer individuals carry a disproportionate burden of financing the minimum health opportunities and, at the same time, they have very unequal access to these basic life-saving services.

In this illustration an important reiteration is that in order to *guarantee* the targeted minimum of health opportunities to all individuals, the whole cost for this minimum should be covered by public resources (contributed by both HIC and LIC governments) so that there will be no PHE for such services, at least at the point of service delivery. With regards to the impact of private health expenditure on health equity, the picture among most LICs is grim. In Uganda, for instance, PHE contributes about 50 % of THE, against 22 and 28 % from domestic public and donor sources respectively [28, 34]. For Rwanda in 2006 these figures were 28, 19 and 53 % respectively [35]; while for Kenya the same figures were around 36.7, 28.8 and 34.5 % for private, public and donor funding respectively for the financial year 2009/10 [36]. Hence, with GINI co-efficiencies as high as 50.8 % for Rwanda in 2011 [37]; 42.5 and 44.3 for Kenya in 2008 and Uganda in 2009 [38] respectively, it means that the higher the PHE as percentage of national THE, the deeper the inequity in health financing. And as long as these domestic inequities remain within LICs, the consequence will remain – as Amouzou and others have discovered – that “economic inequalities with respect to health [U5MR] within developing countries contribute much more to the global health gap than might appear to be the case at first glance; […]” [39]. In this regard it is important to note that reducing health disparities by *economic status* in LICs primarily requires intra-country equity in health financing which reduces the health costs of the poor through cross-subsidies and, therefore, facilitates equal financial access to health services for all. This will be possible if LICs fulfil their minimum financing obligation.

In this paper we have been concerned with global health equity defined as access by everyone to a minimum level of health services. We recommend that the whole cost of the global minimum health opportunities per capita for each country be covered by pooled public resources (domestic plus external resources). Once this has happened there will be no PHE for this minimum, at least at the point of service delivery. Since our current concept of ‘global health equity’ neither targets equality in access to the highest health opportunities globally, nor the same health opportunities for LIC citizens as those of HIC citizens but a rather significantly lower minimum of health opportunities for all individuals, then as long as this minimum is financed equitably there will have existed global health equity as we have defined it.

Certainly there are other health costs other than those at the point of service delivery such as transport costs, time lost in caring for a family member, hours of work lost by patients etc. But all the same the obligation we have proposed will at least ensure equity in health financing at the point of service delivery which mostly hinders access to health services within LICs. With due regard to other sources of health inequities at a domestic level, given that most of these inequities have a financial implications we envisage that promoting and ensuring equity in health financing within LICs is the first necessary step in the right direction which should be followed by other domestic measures. Such measures may include ensuring efficiency, reducing geographical inequities, equitable priority-setting between health interventions (for diseases that mostly affect the poor versus those that affect the rich, or by sex, age etc.) etc., all of which will finally remedy the ills of inequity in global health which arise from domestic contexts. Therefore, given that an argument for this obligation presumes efficiency and equitable mechanism in health resource allocation among others and all of which are not true in most LICs, these two along with other requirements would constitute additional obligations for LIC governments as we have argued elsewhere with respect to general obligations for global justice [40].

In summary, if all LICs are required to allocate a uniform optimal percentage of their domestic budgets to health, fulfilling the resulting obligation by each country and using the resulting deficits to determine the sizes of HIC governments’ obligations to each LIC will have two kinds of effects: the pragmatic effect and fairness effect. From a pragmatic point of view, it will facilitate coordination in global health financing which is necessary to avoid the current duplications arising from uncoordinated international health resource transfers. It will ensure easy monitoring of international health resource flows so that health resources are directed to where they are mostly needed. Its fairness effect consists in ensuring equitable health financing between all countries and within countries for a certain minimum levels of health opportunities for all individuals globally.

#### Potential controversies

One of the potential controversies likely to emerge in implementing LIC obligations in health financing is how to deal with cases where poor countries do not to fulfil their obligations. The first issue is: what should be done if LICs *refuse* [41] to fulfill their obligations? Should the deficits resulting from this refusal be added to HIC obligation for the sake guaranteeing the targeted minimum? We believe that the answer should be no, for at least two reasons. First, it would encourage complacency among LIC governments if there is an automatic mechanism for covering deficits arising from *refusal* to fulfill their obligations. There will be no motivation for LIC governments to increase their domestic budget allocations to health. Instead the motivation might be in the opposite direction since the lesser the domestic resources they allocate to health, the more they would get in international health resource assistance. Rather, what needs to be emphasised here is that the argument for this obligation is based on the WHO premise that “Every country could raise additional domestic funds for health or diversify their funding sources *if they wished to*” (emphasis added), for example, by giving higher priority to health in their domestic budget allocations [15]. Following this premise we hope that it is possible to persuade LIC governments to do so even if it might require some minimum diplomatic nudging. On the other hand, if failure to fulfil their obligations is due to genuine scarcity of resources (*inability*) – say, due to deteriorating economy, unexpected epidemic such as the recent Ebola or war situations etc. which limit the capacity of countries to meet their obligations, then whatever deficits arise from such situations would justly be shared by all parties, or be responded to as a humanitarian situation. The second reason is that requiring HICs to cover deficits which arise out of LIC governments’ *refusal* to fulfill their obligation constitutes injustice against citizens of HICs and will, therefore, make global equity in health financing impossible since HIC citizens would have to contribute more than their proportionate percentage.

The second controversial issue is whether HICs should continue to fulfil their obligations of assistance even if LIC governments refuse to fulfill theirs or, whether international health resource transfers should be construed as rewards to those LICs whose governments fulfill their obligations. We want to emphasise that fulfilling obligations of HICs should not depend on whether LIC governments fulfill their own obligation, nor should international health funds to LICs be seen as a reward for fulfilling their obligations. This is because of at least three reasons: one, whenever they fulfil their obligations, LIC governments are performing their duty for which they do not have to be rewarded. Secondly, international health resources are owed to citizens rather than governments of LICs yet citizens in LICs have no significant influence, if any at all, on budget allocations of their countries like the Uganda example has revealed. Hence, even though governments in LICs refuse to fulfill this obligation their citizens would retain their right to international health resources. The third reason why HICs should continue to fulfill their obligations to LIC citizens even if LIC governments refuse to fulfil their quota of obligations arises from the crucial importance of health in protecting the sanctity of human life and its dignity as well as the centrality of health in ensuring other dimensions of human well-being. Hence, if HICs refuse to fulfill their obligations to the citizens of LICs in such circumstances they would be subjecting the citizens of LICs to a ‘double-jeopardy’. For these reasons, HIC obligations to citizens of LICs should remain morally binding even if LIC governments refuse or fail to fulfil their financing obligations.

## Summary

Achieving justice in global health requires different categories of obligations both by LICs, MICs and HICs. But given that financial resources are pivotal in fulfilling most of these obligations, without overlooking other potential categories of obligations our view is that in order to achieve global health equity it is important to start with ensuring equity in financing global health. Having realised that the current proposals and strategies to raise sufficient resources to that effect largely ignore LIC governments’ obligations, we have proposed, defended and demonstrated the importance of obligations of LIC governments, in particular their obligations regarding minimum health financing. All LICs should allocate to health a certain minimum percentage of their domestically generated annual budget resources as their quota of a global obligation to guarantee to all individuals globally a certain minimum level of health opportunities. The mechanism we have recommended for determining each country’s obligation in covering the cost of minimum health opportunities along with the fulfilment of the resulting obligations will ease coordination in global health fundraising and resource disbursements. This will ultimately lead to global equity in financing a certain minimum level of health opportunities for all individuals globally. However, this obligation can be exploited to wrongly justify withholding international health resources to very poor countries, especially to those which are potential beneficiaries of redistribution but refuse to fulfill their obligations. But given that the obligation to contribute to international health resources is owed to the citizens of LICs not governments *per se*; and given the special nature and value of health in protecting the sanctity of human life and its dignity among others, the failure of governments of LICs to fulfill their obligations should not exempt HIC governments from fulfilling their share of obligations to LIC citizens.

## Abbreviations

GAVI: Global alliance for vaccines and immunisation
GDP: Growth national product
GMHE: Global minimum health expenditure
HIC: High income country
IMF: International monetary fund
LIC: Low income country
LMIC: Low and middle income country
OPP: Out of pocket payment
PHE: Private health expenditure
THE: Total health expenditure
U5MR: Under-five mortality rate
WHO: World health organisation

## Footnotes

1 We recognise that there are many obligations that are necessary to achieve equity in global health but for lack of space we are limiting our current discussion to one of them – equity in financing global health.

2 Even though Ooms and Hammonds are primarily concerned with HIC obligations to give health aid, their analysis implies a belief that LIC governments ought to bear obligations too.

3 What we have done here is a hypothetical illustration. We presume (and recommend) that the process of determining the actual optimal financial obligation of countries and their possible enforcement measures will be evidence-based (especially regarding LICs’ resource capacities), participatory and democratic involving all concerned countries in respect of the principles of national autonomy and sovereignty (and the possible limits of these principles).

4 Even though this argument is centred on obligations of LICs, the mechanism suggested implies that this obligation ought to be borne by all countries that rely on, or need, external resources to reach the GMHE per capita.

5 According to the 2012 *WHO Global health expenditure atlas*, in 2010, within the WHO African region alone, in at least 13 countries PHE for health is more than 50 % of THE with the highest at 88 % in Guinea.
